# Swimming Impedes Intestinal Microbiota and Lipid Metabolites of Tumorigenesis in Colitis-Associated Cancer

**DOI:** 10.3389/fonc.2022.929092

**Published:** 2022-07-01

**Authors:** Weiyi Wang, Ying Xu, Xiaofeng Wang, Yimin Chu, Haiqin Zhang, Lu Zhou, Haijin Zhu, Ji Li, Rong Kuai, Fengli Zhou, Daming Yang, Haixia Peng

**Affiliations:** Department of Endoscopy, Shanghai Tong Ren Hospital, Shanghai Jiao Tong University School of Medicine, Shanghai, China

**Keywords:** chronic inflammation, colonic lipid metabolites, colorectal cancer, intestinal microbiota, physical activity, swimming, tumorigenesis

## Abstract

**Background:**

Accumulating data support that regular physical activity potentially inhibits chronic colitis, a risk factor for colitis-associated cancer (CAC). However, possible effects of physical activity on CAC and the underlying mechanisms remain poorly understood.

**Methods:**

A pretreatment of swimming on azoxymethane/dextran sodium sulfate (AOM/DSS)-induced CAC mice was implemented to determine its protective effect. Inflammation and tumorigenesis were assessed using colorectums from C57BL/6 mice. In order to determine how swimming alters colonic lipid metabolism and gene expression, a comparative analysis was conducted. Meanwhile, alterations in intestinal microbiota and short-chain fatty acids (SCFAs) were detected and analyzed. Finally, an integration analysis of colonic lipid metabolism with gene expression and intestinal microbiota was performed respectively.

**Result:**

Swimming pretreatment relieved bowel inflammation and minimized tumor formation. We demonstrated that prostaglandin E2 (PGE2)/PGE2 receptor 2 subtype (EP2) signaling as a potential regulatory target for swimming induces colonic lipid metabolites. Swimming-induced genera, Erysipelatoclostridium, Parabacteroides, Bacteroides, and Rikenellaceae_RC9_gut_group, induced intestinal SCFAs and affected the function of colonic lipid metabolites enriched in glycerophospholipid metabolism and choline metabolism in cancer.

**Conclusion:**

According to our experiments, swimming pretreatment can protect mice from CAC by intervention in the possible link between colonic lipid metabolites and PGE2/EP2 signaling. Further, swimming-induced genera and probiotics promoted glycerophospholipid metabolism and choline metabolism in cancer, the major constituents of colonic lipid metabolites, and increased SCFAs, which were also important mechanisms for the anti-inflammatory and anti-tumorigenic effects of swimming.

## Introduction

Colorectal cancer (CRC) ranks third among all cancers and as the second leading reason for cancer-related deaths globally (1). By applying early screening programs and developing new treatment regimens, colon cancer has witnessed a dramatic increase in 5-year survival rates. The 5-year survival rate of 91% is achieved for people with localized-stage CRC. However, only 15% of survival rate is achieved for people with metastatic-stage CRC (2). CRC has become a worldwide social and public health problem that imposes enormous humanitarian and financial costs on both the patients, the healthcare system, and society at large.

Healthy Lifestyle Score, comprising five high-potential alterable lifestyle factors (non-smoking, moderate alcohol consumption, a healthy diet, physical activity, and a healthy weight), was correlated with a lower risk of CRC, and the risk was to be further decreased as persistence in a healthy lifestyle score increased (3). A recent large-scale demographic-based study uncovered that a colonoscopy significantly reduces the absolute risk for CRC and that adherence to treatment can further reduce the genetically predetermined risk for CRC (4). Physical activity might protect against about 15% of CRC by its dose-dependent effect, making it an important part of a healthy lifestyle score, and its effect was independent of that of the weight control (5, 6). However, the molecular mechanism involved in the protective effects of physical activity are largely obscure yet.

From cellular changes to systemic metastatic spread, CRC has been undergoing a long development process. We therefore have more chances of discovering the risk factors of CRC and precancerous lesions early, intervening and treating them in time to reverse the development of the tumors. It is widely accepted that chronic inflammation has long been recognized as a crucial trigger of somatic tumorigenesis and progression (7–9). CRC can be categorized as two types: colitis-associated cancer (CAC) and sporadic CRC. Chronic relapsing inflammatory bowel disease (IBD) is an essential risk factor for initiating CAC, which is characterized as 2% of CAC incidence after 10 years and 18% after 30 years with a history of IBD (10, 11). There is opportunity for intraventive measures to halt or even reverse the development of the disease in this high-risk population, in light of the long process from IBD to CAC (12). In a rat model of chronic colitis evoked by the use of dextran sodium sulfate (DSS), Qin et al. found that physical activity treatment (i.e., swimming for 1 or 1.5 h per day, 5 days per week, for 7 weeks) in a dose-determined way prohibits colonic shortening, disruption of the colonic barrier, and splenomegaly. Swimming also alleviated dextran sodium sulfate (DSS)-induced chronic colitis by modulating inflammation, oxidative stress, and apoptosis (13). Additionally, these results may encourage further investigation into potential anti-inflammatory and even anticancer mechanisms associated with swimming.

This study used swimming to simulate the common whole-body aerobic activity people do to investigate whether physical activity prevents AOM/DSS-induced CAC, to research the underlying molecular mechanism, develop strategies for CRC prevention and treatment, improve life quality, and guide practical actions.

## Materials and Methods

### Experimental Animals

Male C57BL/6 mice (age, 4 weeks) from the Animal Science Laboratory, School of Medicine, Shanghai Jiaotong University (Shanghai, China), were purchased. We had pathogen-free mice that are bred and raised in the Animal Care Facility of Tong Ren Hospital, Shanghai Jiaotong University School of Medicine, under laboratory conditions (23°C, 50% humidity, 12/12-h light/dark). Shanghai Tong Ren Hospital, Shanghai Jiao Tong University School of Medicine Ethics Committee authorized an experimental protocol.

### Reagents

Azoxymethane (AOM), dextran sodium sulfate (DSS), and antibody against cyclooxygenase-2 were purchased from Sigma-Aldrich (St. Louis, MO, USA), MP Biomedicals (Santa Ana, CA, USA), and Servicebio Technology (Wuhan, China) respectively.

### Experimental Procedure

**Figure 1:**
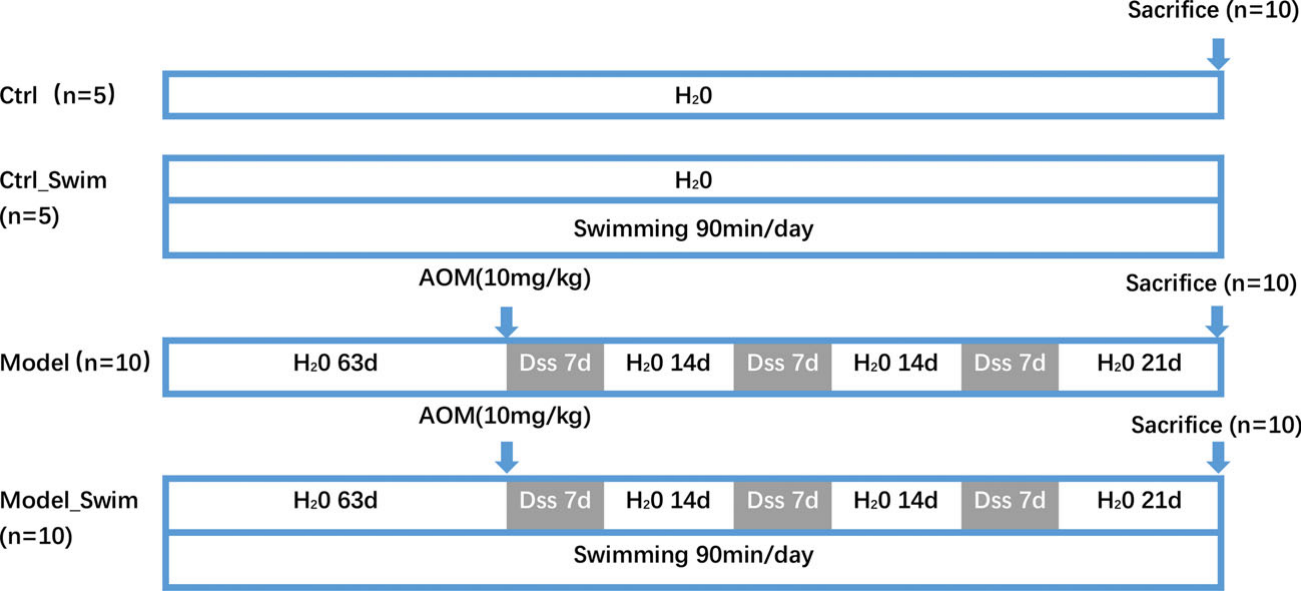
Experimental schedule for AOM/DSS-induced CAC models in mice with swimming pretreatment. Ctrl, control; d, day.

The schedule of the experiment is shown in **Figure 1**. Thirty male C57BL/6 mice were assigned randomly to four groups: the Ctrl group (n = 5), Ctrl_swim group (n = 5), Model group (AOM/DSS only, n = 10), and Model_swim group (swimming while AOM/DSS, n = 10). Projections of AOM/DSS-induced CAC were carried out by our previous study protocol (14). On the first day, a single dose of AOM (10 mg/kg) was injected i.p. to the mice and thereafter 3 cycles of DSS treatment were applied. On each cycle of DSS, animals in the Model and Model_Swimming groups were treated by water with 2% DSS (w/v) continuously for 7 days, then sterile water for 2 weeks. The Ctr and Ctrl_Swim groups, which served as vehicle controls, received ordinary drinking water during the study period.

Mice in the Model_Swim and Ctrl_Swim groups performed swimming as previously described (15), with slight modifications. The mice were left to swim in a tank of 225-cm2 surface area, 15-cm depth, and 30–32°C water temperature. The animals swam initially for 20 min a day, with the swimming time increasing by 10 min per day, until reaching 90 min per day, up to the middle of the second week. After acclimatization, each day (10:00 am) the mice were trained for 2 months before the start of the experiment until the end of the experiment. The untrained mice were placed inside the same glass container containing water (kept up to 35°C) in a 2-cm depth.

### Collection and Preparation of Samples

At the end of the experiment, euthanasia was performed on all animals, and laparotomy was performed immediately. From the distal cecum to the rectum, the colon was resected to remove adherent fatty tissue and dissected longitudinally, and collected feces were stored in -80°C; the colon was washed in ice-cold saline for removing fecal debris, and photographs were taken. Histopathological, immunohistochemical, gene expression, and metabolic investigations were performed by utilizing the isolated colon. Feces were analyzed for 16S rRNA gene sequencing, metabonomic investigation, and SCFA detection.

### Evaluation of Histopathology and Immunohistochemistry

Biopsied colon tissues were checked for tumorigenesis on a macroscopic scale. We fixed the tissues in formalin overnight and subsequently replaced the solution with 70% ethanol before paraffin embedding. H&E staining of paraffin sections was carried out following the criterion processes of histological evaluation (16).

### RNA Isolation and Library Preparation

Total RNA was isolated from the colon tissues using TRIzol Reagent (Invitrogen, Carlsbad, CA, USA) according to the manufacturer’s protocols. Then libraries were then structured using the TruSeq Stranded mRNA LT Sample Prep Kit (Illumina, San Diego, CA, USA) following the manufacturer’s instructions. OE Biotech Co., Ltd. (Shanghai, China), undertook the sequencing and analysis of the transcriptome.

### Analysis of RNA Sequencing and Differentially Expressed Genes

Illumina HiSeq X Ten was utilized to sequence the library. Raw data (raw reads) of fastq format were firstly processed using Trimmomatic (17), and the low-quality reads were removed to obtain the clean reads. The clean reads were mapped to the Mus musculus genome (GRCm38.p6) with HISAT2 (18). Each gene’s FPKM (19), as calculated by Cufflinks (20), and each gene’s read counts were obtained from the HTSeq-count (21). Analysis of differential expression was conducted by the DESeq2 R package (22). Statistically significant differential expression was characterized as q value < 0.05, fold change > 2, or fold change < 0.5. On the basis of hypergeometric distribution, the DEG enrichment analysis was undertaken with R language for Gene Ontology (GO) as well as Kyoto Encyclopedia of Genes and Genomes (KEGG) (23) pathways, respectively. RNA sequencing (RNA-seq) data were deposited in the Gene Expression Omnibus (GEO) of National Center for Biotechnology Information (NCBI) and will be accessible through GEO Series accession number GSE205173 (https://www.ncbi.nlm.nih.gov/geo/query/acc.cgi?acc=GSE205173) after the indicated release date.

### Analysis of the Metabolic Profile

Lumen Biotech (Shanghai, China) employed liquid chromatography–mass spectrometry (LC-MS) for evaluating the lipid metabolism in feces and colonic tissues. When extracting metabolites, the chemical properties of multitarget metabolites require consideration. Lipid metabolites were extracted from 20 mg of feces and 60 mg of tissue with storage at -20°C prior to LC-MS analysis.

A Dionex Ultimate 3000 RS UHPLC fitted with a Q Exactive Plus Quadrupole-Orbitrap Mass Spectrometer equipped with a heated electrospray ionization (ESI) source (Thermo Fisher Scientific, Waltham, MA, USA) was used for analyzing the metabolic profiles in ESI-positive and ESI-negative ion modes. Analysis in positive and negative modes was performed by means of an ACQUITY UPLC HSS T3 column (1.8 μ m, 2.1 × 100 mm). The binary gradient elution system comprised (A) water (with 0.1% formic acid, v/v) with (B) acetonitrile (with 0.1% formic acid, v/v); the following gradients were used to achieve the separation: 5% B, 0 min; 5% B, 2 min; 25% B, 4 min; 50% B, 8 min; 80% B, 10 min; 100% B, 14 min; 100% B, 15 min; 5% B, 15.1 min; and 5% B, 16 min. The flow rate was 0.35 ml/min while the column temperature was 45°C. In the course of the analysis, all samples were stored at 4°C. We set the injection volume to 1 μl, and the mass rises from m/z 100 to 1,000. The resolution of the full mass spectrometry scan at 70,000 and the HCD MS/MS scan at 17,500 was set. The collision energy was set in the ranges of 10, 20, and 40 eV. In accordance with the mass spectrometer, the spray voltage was 3,000 V (−) and 3,800 V (+); the flow rate of sheath gas was 35 arbitrary units; the flow rate of auxiliary gas was 8 arbitrary units; the temperature of the capillary was 320°C; the temperature of the Aux gas heater was 350°C; and the S-lens RF level was 50. Regular injections of quality control (QC) (every 10 samples) were applied throughout the analytical process to demonstrate the reproducibility.

From Lumingbio, the target LC-MS database was used to identify lipid metabolites. Progenesis QI (Waters Corporation, Milford, USA) data processing software was applied to identify metabolites in this study, which was public database based, both http://www.hmdb.ca/ and http://www.lipidmaps.org/ in addition to self-built databases. To visualize metabolic alterations, we utilized principle component analysis (PCA) as well as (orthogonal) partial least square discriminant analysis (O) (PLS-DA). On the combination of a statistically significant variable influence on the projection (VIP) threshold and p values, when VIP > 1.0 and p < 0.05, the differential metabolites were selected.

Short Time sequence Expression Miner (STEM) was employed for identifying significantly different lipid metabolites (p < 0.05). Different-metabolite pathway enrichment was analyzed with the KEGG database. With these data, a gene–pathway–metabolite network was constructed by Cytoscape.

### Short-Chain Fatty Acid Analysis

Short-chain fatty acids (SCFAs) of feces (20 mg) were extracted. Afterward, samples were placed at -20°C for at least 30 min, then a 0.22-μm organic-phase pinhole filter was applied through for subsequent ultra-performance LC (UPLC)-tandem MS (MS/MS) analysis by Luming Biotech Ltd. (Shanghai, China).

### Sequencing Analysis of the 16S rRNA Gene

Fecal samples from experimental mice were extracted for DNA with a QIAamp Fast DNA Stool Mini Kit (Cat# 51604, QIAGEN, Venlo, Netherlands). Amplification of bacterial DNA was carried out by primers targeting the V3–V4 regions (5′-TACGGRAGGCAGCAG-3′, 5′-GGGTATCTAATCCT-3′). OE Biotech in Shanghai conducted DNA sequencing with a MiSeq PE300 platform from Illumina (CA, USA).

Trimmomatic software (17) is an application of preprocessing paired-end reads for detecting and cutting off obscure base(s) (N). In addition, it trimmed the quality score below 20 for low-quality sequences using the trimming method for sliding windows. Following trimming, FLASH software assembled the paired-end reads (24). The assembly parameters are as shown below: minimum overlap of 10 bp, maximum overlap of 200 bp, with a 20% maximum mismatch rate. We performed further denoising of the sequences in the following details: dropping reads with obscure, homologous sequences or less than 200 bp. Utilizing QIIME software (version 1.8.0), only 75% of the reads for bases above Q20 were retained (25). Followed by using VSEARCH, reads with chimeras were detected and removed (26). VSEARCH software was used to generate operational taxonomic units (OTUs) by clustering with a cutoff of 97% similarity. An RDP classifier was used to annotate the OTU against version 123 of the Silva database (70% confidence threshold) using QIIME software (27). In fecal samples, microbial diversity analysis was performed by the Chao1 index (28) and Shannon index (29). A binary Jaccard-based principal coordinate analysis (PCoA) was carried out by using the UniFrac distance matrix generated by QIIME software. Microbial multivariate statistical analysis used Kruskal–Wallis difference statistics. The bacterial species that differ among the four groups were identified by linear discriminant analysis and effect size measurements (LEfSe). OE Biotech Co., Ltd. (Shanghai, China), performed the sequencing and analysis of 16S rRNA. The 16S sequencing data were deposited in the NCBI BioProject under accession number PRJNA845074. The Sequence Read Archive (SRA) records will be accessible with the following link after the indicated release date: http://www.ncbi.nlm.nih.gov/bioproject/845074.

### Statistics

By utilizing GraphPad Prism 9.0 software, Student’s t tests were employed in analyzing gene expression, tumors number, tumors size on average, colon length, and bacterial diversity. Significant results were considered if 2-tailed p-values <0.05.

## Results

### Swimming Pretreatment Attenuates AOM/DSS-Induced Tumor Development

**Figure 2:**
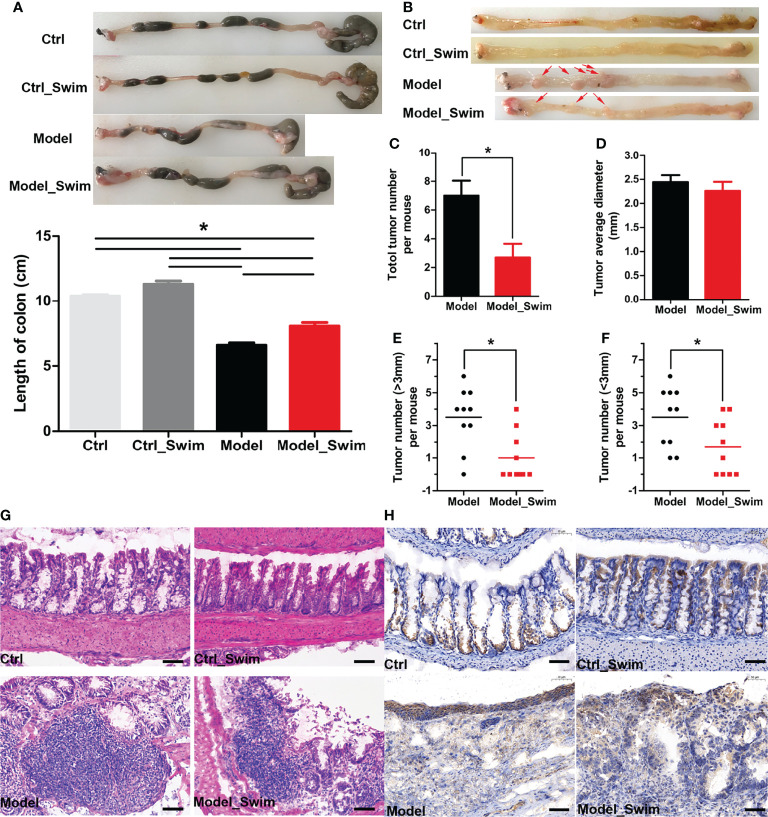
Pretreatment with swimming alleviates colitis and tumorigenesis in AOM/DSS-induced CAC mouse models. **(A)** Representative images for both colon morphology and colonic length statistics. **(B)** Representative macrographic impressions. Remarkable red arrows represent tumors larger than 1 mm diameter. **(C–F)** Tumor morphology, tumor diameter and tumor distribution statistics. **(G)** The representative colonic H&E section. **(H)** Stained immunohistochemically by Cyclooxygenase-2 (COX-2). Results were presented by mean ± SEM. *P < 0.05, compared with Model group. Original magnification, ×200.

The chemopreventive effect of swimming on CAC evaluated. The shortening of colonic length was significantly alleviated after swimming pretreatment, which indirectly suggested that swimming had chemopreventive effect on colitis (**Figure 2A**). We then focused on observing the neoplastic lesions on AOM/DSS induced CAC mice, and the macroscopic images of the colon in different groups of mice were shown in **Figure 2B**. Most tumors were located in the colorectum ranging from 1 mm to 5 mm, and a few were in the proximal colon. Swimming pretreatment significantly inhibited colitis-induced tumor’s multiplicity and size, by a 60% tumorigenicity rate and an average of 2.7 gross tumors (mean diameter, 2.25 mm) each mouse of Swimming-pretreated mice vs a 100% tumorigenicity rate and 7 tumors (mean diameter, 2.45 mm) each mouse of the Model group (**Figures 2C–F**). In histological assessment, mice of the Model group displayed multiple adenomas and invasive adenocarcinomas, while mice of the Model_Swim group were characterized by disease mainly in the form of crypt dysplasia and adenomas (**Figure 2G**). In addition, swimming affected the expression of cyclooxygenase-2 (COX-2) in the colon. The expression of COX-2, one of inflammation-related enzyme and exercise-induced expression alteration biomarkers (9, 30), was upregulated in mucosal surfacing by AOM/DSS in Modle group (**Figure 2H**, Model**)**, and was down in Swimming pretreatment group (**Figure 2H**, Model_Swim**).**

### AOM/DSS and Swimming Pretreatment-Induced Changes in Transcription

**Figure 3:**
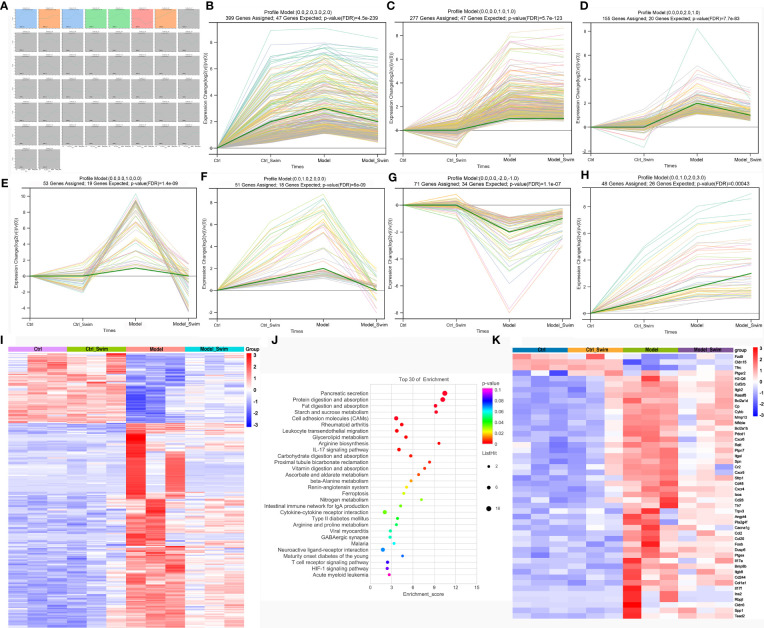
Variations in transcription induced by AOM/DSS and swimming pretreatment. **(A–H)** Patterns of gene expression across the four groups inferred by Short time−series expression minor clustering analysis (STEM) analysis. **(I)** Heatmap of the differential genes caused by swimming in AOM/DSS-induced CAC Mice. **(J)** KEGG enrichment analysis of the differential genes caused by swimming in AOM/DSS-induced CAC Mice. **(K)** Heatmap of the screened 48 differential genes caused by swimming in AOM/DSS-induced CAC Mice.

We compared gene transcript expression between groups and selected the concatenation of differential genes, i.e. 1403 genes differentially expressed genes for subsequent analysis. Short Time-series Expression Miner (STEM) was applied for analysis of 1403 differential genes (**Figure 3A**), and we considered that the differential expression of the genes, which were elevated or decreased in the Model group compared to the Ctrl and Ctrl_Swim groups and could be correspondingly decreased and increased in the Model_Swim group were influenced by swimming. In all, 1054 out of 1403 of the differentially expressed genes were significantly clustered in seven models, including profile 49 (**Figure 3B**), profile 29 (**Figure 3C**), profile 30 (**Figure 3D**), profile 28 (**Figure 3E**), profile 41 (**Figure 3F**), profile 22 (**Figure 3G**) and profile 42 (**Figure 3H**), only 279 genes of profile 30 (**Figure 3D**), profile 28 (**Figure 3E**) and profile 22 (**Figure 3G**) met our screening criteria and were screened (**Figure 3I**). KEGG enrichment analysis of the 279 genes showed many functions involving digestion and absorption, metabolism of nutrient elements, and about adhesion, migration, and cytokine interactions (**Figure 3J**), suggesting that swimming motivates body nutrient utilization and energy metabolism as well as acts as an inhibitor of colon cancer by affecting the gene expression of several aspects of inflammatory immune-related functions in AOM/DSS-induced CAC mice. We therefore selected these functionally enriched inflammatory immune-related genes which are directly related to tumorgenesis for analysis, a total of 48 differential genes were involved (**Figure 3K**). Among which we observed that only three genes expression, Fzd9, Cldn15 and Tfrc in the Model group were the lowest, while the other genes expression in each group were the opposite. Meanwhile, we observed that the AOM/DSS induced CAC mice upregulated Ptger2, Pla2g4f and Ptges expression, to increase synthesis of Prostaglandin E2 (PGE2) receptor 2 subtype (EP2), a G protein-coupled receptor, and that PGE2, a downstream arachidonic acid metabolites of COX-2 and prostaglandin E synthase (PTGES), binds to EP2 to mediate multiple intracellular signaling pathways to promote colon cancer development (9, 31, 32). The rest of the genes were mainly related to Cell adhesion molecules (CAMs), Leukocyte transendothelial migration, IL-17 signaling pathway, Ferroptosis, Intestinal immune network for IgA production, Cytokine-cytokine receptor interaction, etc. Swimming may inhibit CAC processes by inhibiting PGE2/EP2 receptor-ligand binding and intracellular signaling pathways mentioned above.

### Swimming Pretreatment Alters Composition of the Intestinal Microbiota Community

Overall, 16S rRNA sequencing produced 57,318 and 71,203 valid tags of average lengths 271.43 to 440.29 bp. In our result, we got 4666 operational taxonomic units (OTUs) that were part of 16 phyla, 24 classes, 73 orders, 119 families, and 246 genera. The species accumulation curve and the rarefaction curve(**Supplementary Figure. S1**) from a total of all samples reflected that the sequencing depth was adequate to characterize the bacterial richness and diversity of all samples. Following false discovery rate (FDR) correction (FDR < 0.05), 1606 OTUs, 0 phyla, 1classes, 12 orders, 23 families, and 55 genera were significantly different among the 4 groups.

**Figure 4:**
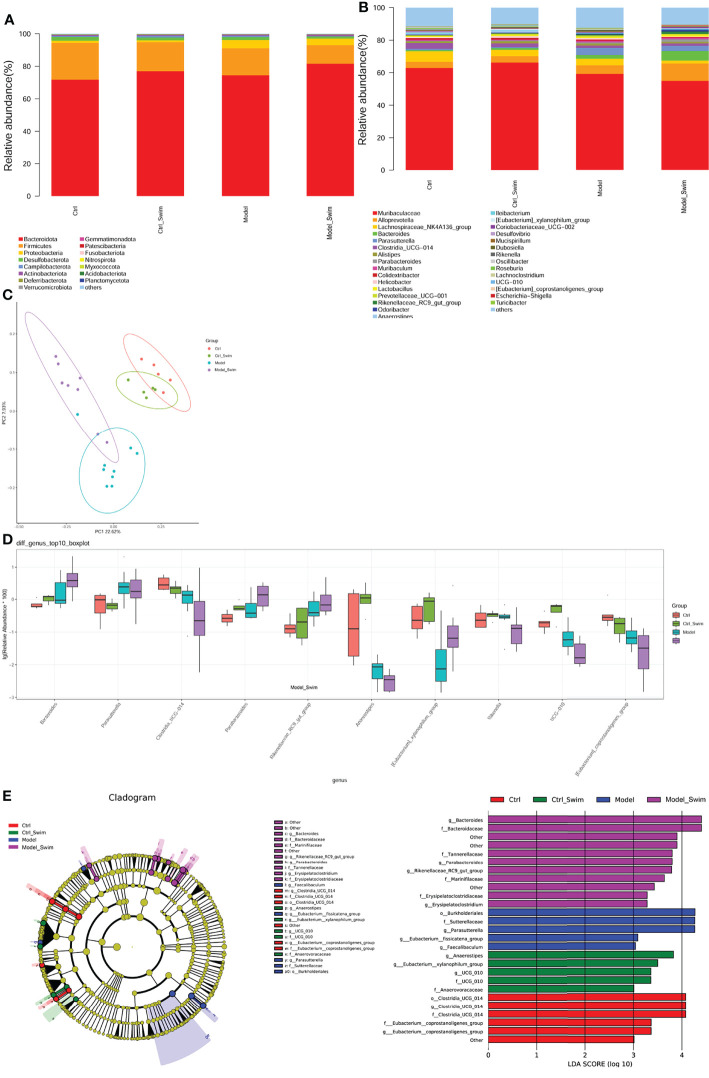
Alteration of fecal microbial community composition caused by swimming pretreatment in AOM/DSS-induced CAC Mice. **(A)**, Relative abundance in each group for the major bacterial phylum. **(B)**, Relative abundance of the main bacterial genus in each group. **(C)**, Fecal microbial community diversity analyses. Binary jaccard-based PCoA plot base of the relative abundance of OTUs displaying bacterial structural clustering. **(D)**, The top 10 genera with significant differences for relative abundances among the Model, Model_Swim, Control and Control_Swim groups; Kruskal–Wallis, all p < 0.05. **(E)**, Analysis of the different species composition among the Model, Model_Swim, Control and Control_Swim groups by LEfSe.

At the phylum level, Bacteroidota increased after swimming (Ctrl_Swim:76.98%, Model_Swim:81.55% vs Ctrl: 71.77%, Model: 74.39%) and Firmicutes decreased (Ctrl_Swim:17.78%, Model_Swim:11.39% vs Ctrl:22.59%, Model:16.63%) (**Figure 4A**). At the genus level, the Model_Swim group had the lowest percentage of Muribaculaceae, Lachnospiraceae_NK4A136_group, Colidextribacter and the highest percentage of Alloprevotella, Bacteroides, Parabacteroides among the four groups, and it was also observed that Lactobacillus, a related genus in our previous study (14), had the highest percentage among the four groups (**Figure 4B**).

Although Chao1 estimates and Shannon index did not differ significantly among the groups(**Supplementary Figure S2**), however, binary jaccard-based PCoA analysis combined with the results of adonis analysis (P=0.001), demonstrated significant differences in beta diversity among the groups (**Figure 4C**). In further multivariate statistical analysis, Kruskal Wallis difference statistics identified 55 differential genera, of which Bacteroides, Parabacteroides, Rikenellaceae_RC9_gut_group were significantly more abundant in the Model_Swim group than the other three groups, while Clostridia_UCG-014, Anaeroplasma, Rikenella, UCG-010, [Eubacterium]_coprostanoligenes_group were significantly less abundant in the Model_Swim group than the other three groups (**Figure 4D** and **Supplementary Table S1**).

A cladogram generated by Linear discriminant analysis coupled with effect size measurements (LEfSe) was used to identify the specific bacteria linked to swimmimg (**Figure 4E**). The result shown that Erysipelatoclostridium, Parabacteroides, Bacteroides, Rikenellaceae_RC9_gut_group were significantly dominant (LDA scores (log10) >3.0) in the Model_Swim group, while Faecalibaculum, Parasutterella, Eubacterium:fissicatena_group were the most abundant in the Model group (LDA scores (log10) >3.0). Together, these results suggest that changes in the intestinal microbiota composition were linked to swimmimg.

### Lipid Metabolism Changes in Colonic Environment Induced by AOM/DSS and Swimming Pretreatment

The transcriptome sequencing and analysis showed that transcriptome functions were enriched to many digestion and absorption, nutrient metabolism, which hinted the effects of swimming on these aspects. As a natural lipids, the biosynthesis of PGE2 is inhibited by swimming, which attract us to investigate how swimming affects lipid metabolism in the colonic environment, which include colonic tissue and direct contacted feces, to inhibit PGE2 synthesis and its functional signaling pathway.

**Figure 5:**
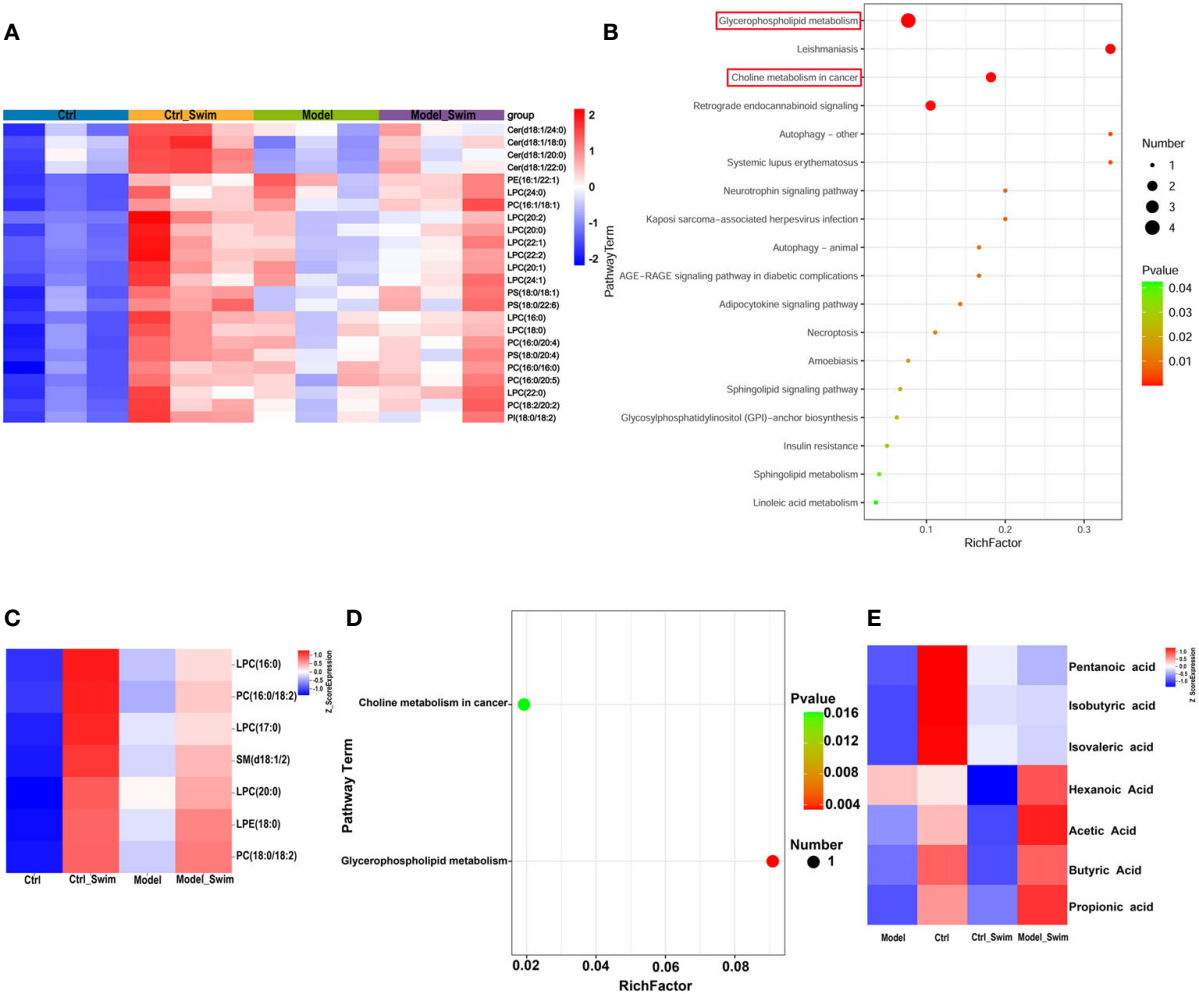
Lipid metabolism changes in colonic environment induced by AOM/DSS and swimming pretreatment. **(A)** Heatmap of 24 differential colonic lipid metabolites with functional enrichment. **(B)** KEGG enrichment analysis of the differential colonic lipid metabolites caused by swimming in AOM/DSS-induced CAC Mice. **(C)** Heatmap of 7 differential fecal lipid metabolites caused by swimming in AOM/DSS-induced CAC Mice. **(D)** KEGG enrichment analysis of the differential fecal lipid metabolites caused by swimming in AOM/DSS-induced CAC Mice. **(E)** Heatmap of fecal SCFAs alteration by swimming in AOM/DSS-induced CAC Mice.

Firstly, we analyzed lipid metabolites in colonic tissue. We used PCA and (orthogonal) partial least squares discriminant analysis (OPLS-DA), and separate between Ctrl & Ctrl-Swim, Ctrl & Model, Ctrl-Swim & Swim, and Swim & Model, respectively. We separate between Ctrl and Ctrl_Swim (R2Y (cum) = 0.835 and Q2 (cum) = 0.598), Ctrl and Model (R2Y (cum) = 0.788 and Q2 (cum) = 0.642), Ctrl_Swim and Model_Swim (R2Y (cum) = 0.622 and Q2 (cum) = 0.82), and Model_Swim and Model (R2Y(cum) = 0.788 and Q2 (cum) = 0.642), respectively. Which demonstrated the presence of metabolic differences in these two groups. Excellent stability and reproducibility of the analytical platform were displayed by the permutation test (Ctrl & Ctrl_Swim, R2 = 0.965, Q2 = -0.137; Ctrl & Model, R2 = 0.959, Q2 = -0.16; Ctrl-Swim & Model_Swim, R2 = 0.974, Q2 = -0.151; Model_Swim & Model, R2 = 0.965, Q2 = 0.002), which can be employed in the follow-up metabolomics research (**Supplementary Figure S3**). Total of 685 metabolites were found. According to the STEM analysis (**Supplementary Figure S4**), 233 differential metabolites were found among the four groups, which decreased in the Model group but rebounded in the swimming groups(**Supplementary Figure S5**). The KEGG pathway enrichment analyses of 233 differential metabolites indicated that a total of 24 differential lipid metabolites with functional enrichment (**Figure 5A**) were related to Glycerophospholipid metabolism, Leishmaniasis, Choline metabolism in cancer, Retrograde endocannabinoid signaling, Autophagy – other, Systemic lupus erythematosus Neurotrophin signaling pathway, Kaposi sarcoma-associated herpesvirus infection, AGE-RAGE signaling pathway in diabetic complications, Autophagy – animal, Adipocytokine signaling pathway, Necroptosis, Amoebiasis, Sphingolipid signaling pathway, Glycosylphosphatidylinositol (GPI)-anchor biosynthesis, Insulin resistance, Sphingolipid metabolism, Linoleic acid metabolism, alpha-Linolenic acid metabolism, Glycine, serine and threonine metabolism, and Arachidonic acid metabolism (**Figure 5B**).

Secondly, the fecal metabolome is considered as an intestinal microbiome functional readout. Fecal metabolic analysis as a new tool to explore the connection of microbiota composition with the host phenotype (33). As described in colonic lipid metabolism analysis, PCA, OPLS-DA, and permutation test were performed for quality control of fecal lipid metabolites analysis (**Supplementary Figure S6**). In total, 75 differential metabolites were observed in fece. On the base of a differentiated screening strategy for STEM analysis (**supplementary Figure S7**), 7 discriminating metabolites were found among the four groups, which were also significantly highly expressed in the swimming groups (**Figure 5C**). The KEGG pathway enrichment analyses revealed that these differential metabolites were involved in Glycerophospholipid metabolism and Choline metabolism in cancer (**Figure 5D**).

In addition, we performed SCFAs UPLC-MS/MS analysis on mouse feces and showed that SCFAs of the Model_Swim group was higher than that of the Model group, and the expression of Propionic Acid, Butyric Acid and Acetic Acid was higher in the Ctrl group and the Model_Swim groups, but lower in the Model group, especially Butyric Acid (P<0.05) (**Figure 5E**).

### Integration Analysis Among Intestinal Microbiota Community, Colonic Lipid Metabolites, and Transcriptomics to Construct Anti-Inflammatory and Anti-Tumorigenic Transcriptional Networks

**Figure 6:**
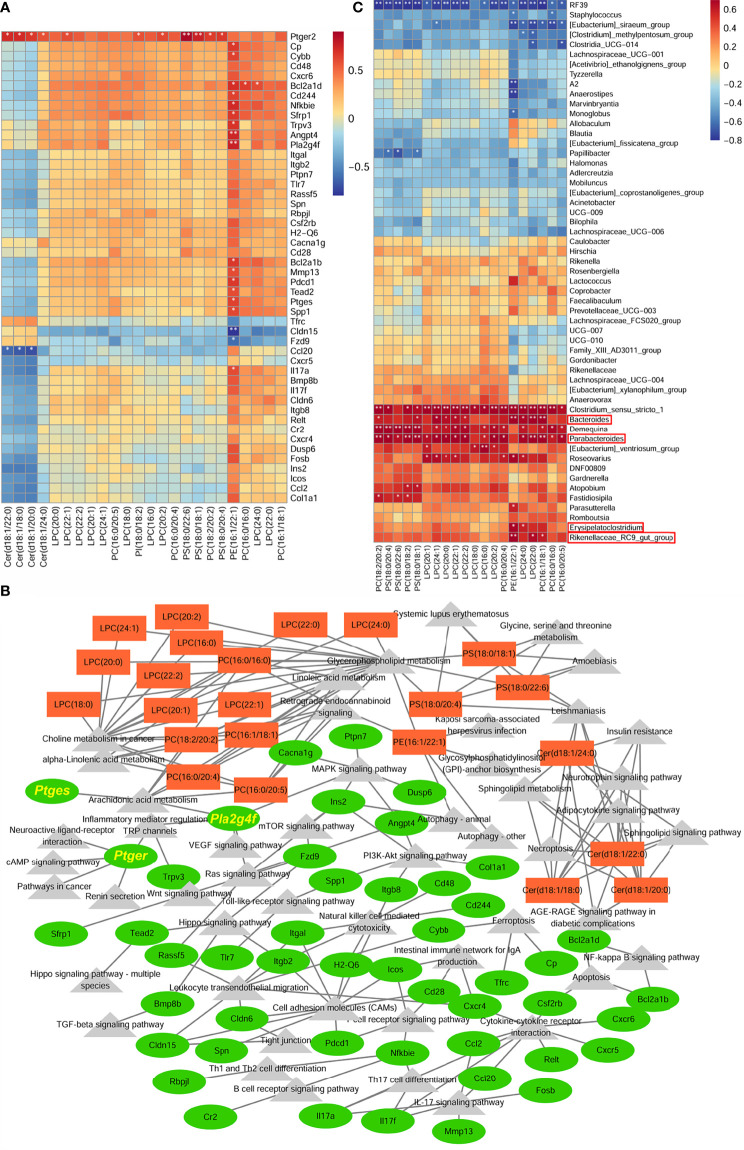
Integration analysis between colonic lipid metabolites and transcriptomics to construct anti-inflammatory and anti-tumorigenic transcriptional network. **(A)** Heatmap of correlation between 24 colonic differential lipid metabolites with functional enrichment and 48 differentially expressed genes. **(B)** Correlation network analysis between differential genes and metabolites. **(C)** Heatmap of correlation between colonic differential lipid metabolites, functionally enriched in Glycerophosphlipid metabolism and Choline metabolism in cancer, and the differential fecal microbiota. *P < 0.05, **P < 0.01.

Since the development of CRC is influenced by the interaction of the tumor microenvironment (TME) and the intestinal microbiota (34). In the present experiments, the changes induced by swimming, both systemic within the organism and changes in the intestinal flora, and their effects on the TME are directly reflected in the alteration of colonic lipid metabolites. A Spearman correlation analysis was performed to analyze the covariation between 24 colonic differential lipid metabolites with functional enrichment and 48 differentially expressed genes we selected (**Figure 6A**). The results showed that colonic lipid metabolites: PS(18:0/22:6), PS(18:0,18:1), PS(18:0,18:1), PC(18:0/20:4), PC(18:2/20:2), LPC(22:1), LPC(20:2), Cer(d18:1/18:0), Cer(d18:1/20:0), Cer(d18:1/22:0), Cer(d18:1/24:0) were closely related to Ptger2 expression, where PE(16:1/22:1) was closely associated with many gene expressions (e.g. Pla2g4f, Ptges). Cytoscape showed that the metabolites and gene functions were co-enriched for “arachidonic acid metabolism”, suggesting that colonic lipid metabolites potentially inhibit inflammatory-cancer development by suppressing Ptger2, Pla2g4f and Ptges expression, PGE2/EP2 ligand/receptor binding and activation of intracellular related signaling (**Figure 6B**).

In addition, differential metabolites in fecal and colonic tissue were functionally co-enriched in Glycerophosphlipid metabolism and Choline metabolism in cancer, which means swimming induced dominant genera mainly regulate metabolites of these two functions to exert anti-inflammatory and anti-tumorigenic effects. The correlation analysis between the differential intestinal microbiota and colonic differential lipid metabolites showed that colonic differential metabolites were positively associated with Erysipelatoclostridium, Parabacteroides, Bacteroides, Rikenellaceae_RC9_gut_group (dominant genera in the Model_Swim group) (**Figures 6C**).

In short, colonic lipid metabolites may play an anti-inflammatory and anti-cancer role by inhibiting PGE2/PTGER2 ligand/receptor binding and activating intracellular related signaling, while the metabolites with a major function in Glycerophosphlipid metabolism and Choline metabolism in cancer are influenced by swimming-induced flora alterations.

## Discussion

Chronic inflammation is a recognized risk factor for CRC (9). The previous study showed that swimming attenuates DSS-induced chronic colonic inflammation by regulating inflammation, oxidative stress and apoptosis (13). Our research showed that swimming reduced neoplasia formation rate and tumor number in mice model of AOM/DSS-induced CAC. After being shown to have anti-inflammatory effects, swimming has again been shown to have anti-inflammatory and tumorigenesis prevention effects by animal models. While exhibited anti-inflammatory effects of swimming in the transcriptional dimension by modulating numerous inflammatory signaling pathways. Our experiments also showed that the expression of COX-2, PTGES, Group IV phospholipase A2 family (PLA2G4F) and EP2 were elevated in the model group. PLA2G4F, COX-2 and PTGES are important enzymes for the synthesis of its downstream bioactive lipid products, PGE2, which binds to EP2, and act as multiple signaling pathways of modulating various pathophysiological functions in the tumor microenvironment (TME), including chronic inflammation, invasion and metastasis, cell apoptosis angiogenesis, tumor immune evasion and tumor occurrence (31, 32). In contrast, the increasing of these PGE2/EP2 pathway markers were reduced in the swimming groups. In the latest decades, COX-2 and its prostaglandin products are attracting growing interest due to their important role in colon cancer as well as in other tumors progression. However, the use of nonsteroidal anti-inflammatory drugs (NSAIDs) to inhibit COX-2 or specific COX-2 inhibitors has caused various side effects, including peptic ulcer and its complications and cardiovascular events (35, 36). These have limited the use of these drugs. The above animal experimental phenomena, consistent with preclinical studies (30), suggest that swimming may play an anti-inflammatory and tumorigenesis prevention role, and it is more acceptable to the general public and has no significant side effects risk.

Metabolic reprogramming is an important hallmark of cancer pathogenesis (37, 38). In our model, we observed that swimming reduces tumorigenicity in mice by modulating bioactive lipid products, Prostaglandin E2 (PGE2). It is suggested that lipid metabolites, as energy reserves and signaling transmitters, have a vital role in this process. The Swimming-induced changes of colonic lipid metabolites in CAC was mainly observed. We found that swimming induced the expression of 24 functionally enriched colonic lipid metabolites and regulated the expression of numerous genes closely associated with tumor development, and significant correlation between the metabolites and the genes were displayed by Correlation analysis and Cytoscape. These findings provide evidence for further investigation of the relevance of swimming-induced colonic lipid metabolites to tumors and their pathophysiological mechanisms.

With the researches on physical activity and diseases, we can easily relate to obesity, metabolic diseases prevention and control, for example, metabolic syndrome, type 2 diabetes, cardiovascular disease, etc. Researches had found that physical activity plays a role in preventing and reversing diseases by controlling inflammation, body weight and reducing insulin resistance (39, 40), however, the role in cancer prevention is not related to the body mass index (BMI) (41). Intensive studies of numerous physical activity intervention trials suggested a substantial association with physical activity and tumors development by exercise induced systemic alterations, such as endocrine system, immune response, metabolic changes (42–45). TME is a mixture of stromal and immune cells that determine cancer progression and influence response to tumor therapy, tumor-derived PGE2 undermines immunity against tumors via targeting natural killer (NK) cells, conventional type 1 dendritic cells (cDC1) (35). Besides, physical activity leads a rapid surge of catecholamines in the blood and an increasing influx of various immune cells into the tumor, e.g. NK cell, cDC1 (46). The altered lipid metabolite expression induced by swimming in our study was closely associated with inhibition of PGE2/EP2 signaling pathway, suggesting that these lipid metabolites exert anti-tumor effects by inhibiting immune escape caused by PGE2. Our results partially refine the mechanism of physical activity against cancer from a metabolic perspectives. Among these lipid metabolites, it is possible to screen for lipid metabolites with significant anti-inflammatory and tumoriogenesis prevention effects and potential to become pharmaceutical target for cancer treatment. As well as exploring the role of lipid metabolites as markers for clinical application in the diagnosis, staging and prognostic prediction of CRC.

There is a mutual and highly dynamic communication between tumor cells and TME that strongly influences all steps of tumorigenesis, moreover TME and tumors also have a systemic interaction with the whole organism, including the microbiota (47). The intestinal microbiota is a component of the formation and development of CRC along with its response on therapy. Several studies have attempted to reduce CRC risk by Dietary interventions, Weight reduction, Administration of probiotics (48). As one of the microbiota-derived metabolites, physical activity also increases fecal short-chain fatty acid (SCFAs, e.g. butyrate and propionate) which are produced from non-digestible carbohydrates in the gut, for instance, fiber and resistant starch, by the fermentation of intestinal microbiota (49–51). As an anti-inflammatory molecule, butyrate and propionate deregulate both pro-inflammatory cytokines, induce apoptosis in CRC cell lines, and modulate colonic regulatory T (Treg) cells to exert effective anti-inflammatory efficacy in animal models (52–57).. In our study, swimming changes the ratio of Firmicutes/Bacteroidetes, β-diversity, and LEfSe analysis showed significantly high abundance of 4 genera in Model_Swim group. Among the dominant genera, several species of Parabacteroides and Bacteroides supply of nutrients and vitamins both to the host and to other intestinal microbial inhabitants (58, 59). Which could partially explain why the content of several SCFAs in Model_Swim group higher than in Model group. These findings were also coherent to our previous preclinical research (14) suggesting that swimming exerts anti-inflammatory and tumorigenesis prevention role by altering intestinal microbiota and increasing SCFAs levels as oral supplementation. In addition, we identified seven additional lipid metabolites from feces that increased after swimming, which were functionally co-enriched with tissue-derived lipid metabolites in two functional signaling pathways, Glycerophosphlipid metabolism and Choline metabolism in cancer, suggesting that swimming-induced dominant genera were involved in anti-inflammatory and tumorigenesis prevention effects via these two functional pathways. Further correlation analysis of swimming-induced dominant genera with colonic lipid metabolites enriched in these two functions showed significant relevance further supporting this scenario.

Within contemporary exercise science, mice are widely employed to discover exercise intervention mechanisms for disease prevention. There are still limitations to the mouse model, however. First, mice do not mimic certain forms of movement in humans, but the swimming is the easiest kind of systemic aerobic exercise to simulate. Second, the CAC-induced mouse model does not accurately capture the pathophysiological processes of CRC in human. Third, the functional database of lipid metabolites is not yet complete, and the functions of different lipid metabolites are not fully enriched, and only a small part of the anti-inflammatory and carcinogenesis prevention functional mechanisms of lipid metabolites can be explained by the existing database. Further research as well as optimization of the metabolite functional database would be necessary.

As a conclusion, we demonstrated that the swimming pretreatment relieved AOM/DSS-induced CAC, by exerting anti-inflammatory and tumorigenesis prevention functions. Positive impact of swimming were relevant to its capacity to regulate PGE2/EP2 signaling pathway by modifying colonic lipid metabolites. The swimming can increase SCFAs in the intestine by altering the intestinal microbiota, such as increasing abundance of probiotics, and the efficacy was exerted by swim-induced genera affecting the function of colonic lipid metabolites enriched in Glycerophosphlipid metabolism and Choline metabolism in cancer. These findings demonstrated swimming was a potent preventive measure against CAC, and implied that the differential lipid metabolites screened in the experiment were candidates of medicine with anti-inflammatory and tumorigenesis prevention properties. However, the exact mechanism of the beneficial effects attributed to the lipid metabolites still deserve to be elucidated. Therefore, the research should need further screening of highly efficacious anti-inflammatory and tumorigenesis prevention lipid metabolites and intensively study their molecular mechanisms, as well as screening of tumor markers for colon tumors.

## Data Availability Statement

RNA sequencing (RNA-seq) data were deposited in the Gene Expression Omnibus (GEO) of National Center for Biotechnology Information (NCBI) and will be accessible through GEO Series accession number GSE205173 (https://www.ncbi.nlm.nih.gov/geo/query/acc.cgi?acc=GSE205173) after the indicated release date. The 16S sequencing data were deposited in the NCBI BioProject under accession number PRJNA845074. The Sequence Read Archive (SRA) records will be accessible with the following link after the indicated release date: http://www.ncbi.nlm.nih.gov/bioproject/845074.

## Ethics Statement

The animal study was reviewed and approved by The Ethical Committee of Tongren Hospital, Shanghai Jiaotong University School of Medicine. Written informed consent was obtained from the owners for the participation of their animals in this study.

## Author Contributions

WW designed the experiment. YC, HQZ, LZ, HJZ, JL, RK, and FZ performed the experiment. YX and XW processed the data. WW wrote the paper. DY and HP revised the paper. All authors approved the final version to be published and agree to be accountable for all aspects of the work.

## Funding

This work was supported by Shanghai Municipal Natural Science Foundation (21ZR1458600), Interdisciplinary Program of Shanghai Jiao Tong University (YG2022ZD031), Scientific research project of Health and Wellness Committee Changning District Shanghai (20214Y007), the research Fund of Key laboratory for translation research and innovative of gastrointestinal oncology (ZDSYS-2021-01), and Science and Technology Commission of Changning District of Shanghai (No. CNKW2018Y02).

## Conflict of Interest

The authors declare that the research was conducted in the absence of any commercial or financial relationships that could be construed as a potential conflict of interest.

## Publisher’s Note

All claims expressed in this article are solely those of the authors and do not necessarily represent those of their affiliated organizations, or those of the publisher, the editors and the reviewers. Any product that may be evaluated in this article, or claim that may be made by its manufacturer, is not guaranteed or endorsed by the publisher.
