# Supplementary figures and images for: Swimming Impedes Intestinal Microbiota and Lipid Metabolites of Tumorigenesis in Colitis-Associated Cancer
Source: Front Oncol. 2022 Jul 1;12:929092. doi: 10.3389/fonc.2022.929092 (PMC9285133; doi:10.3389/fonc.2022.929092)

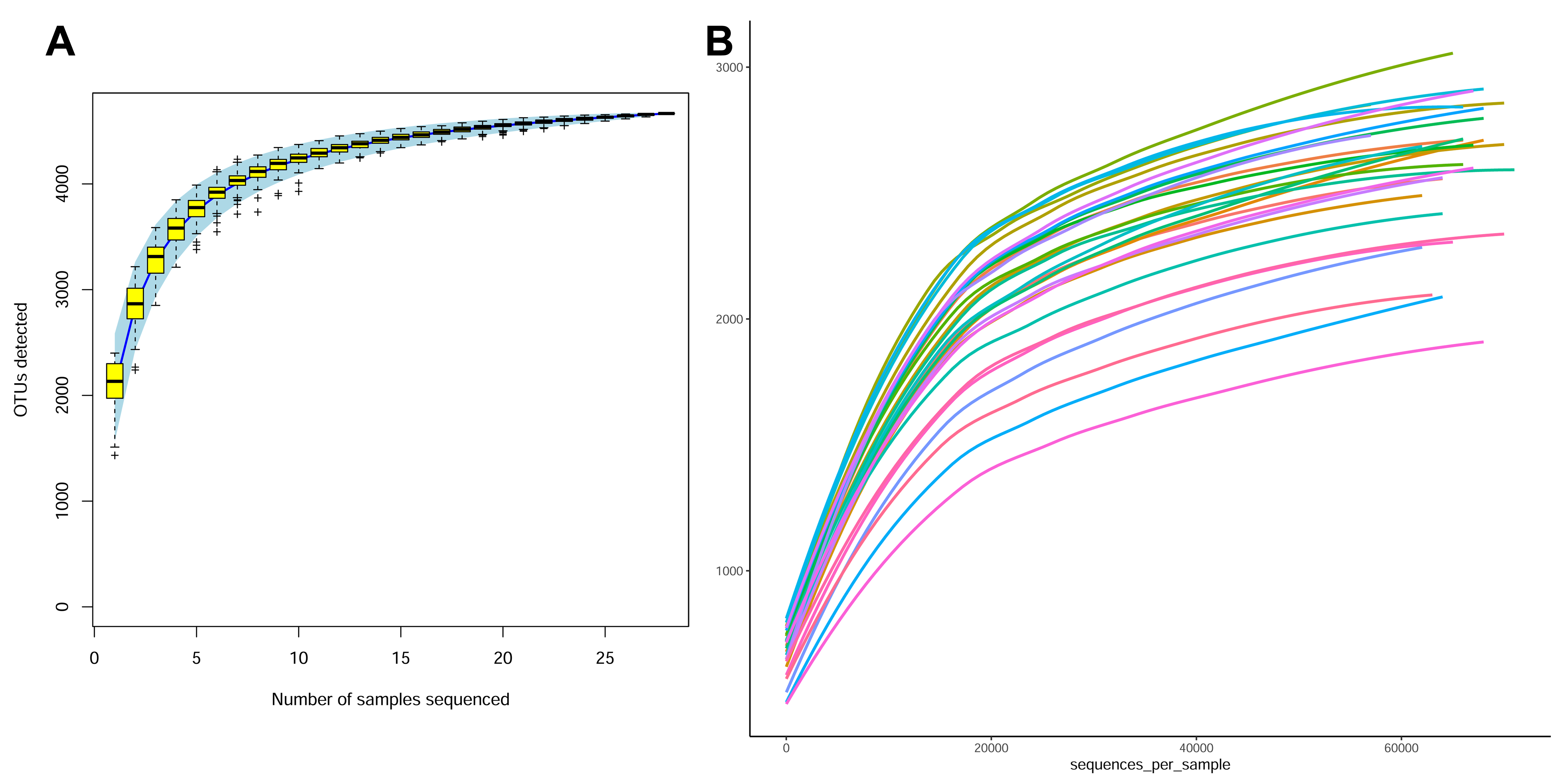

Supplement: Supplementary file 1 [file Image_1.tif]

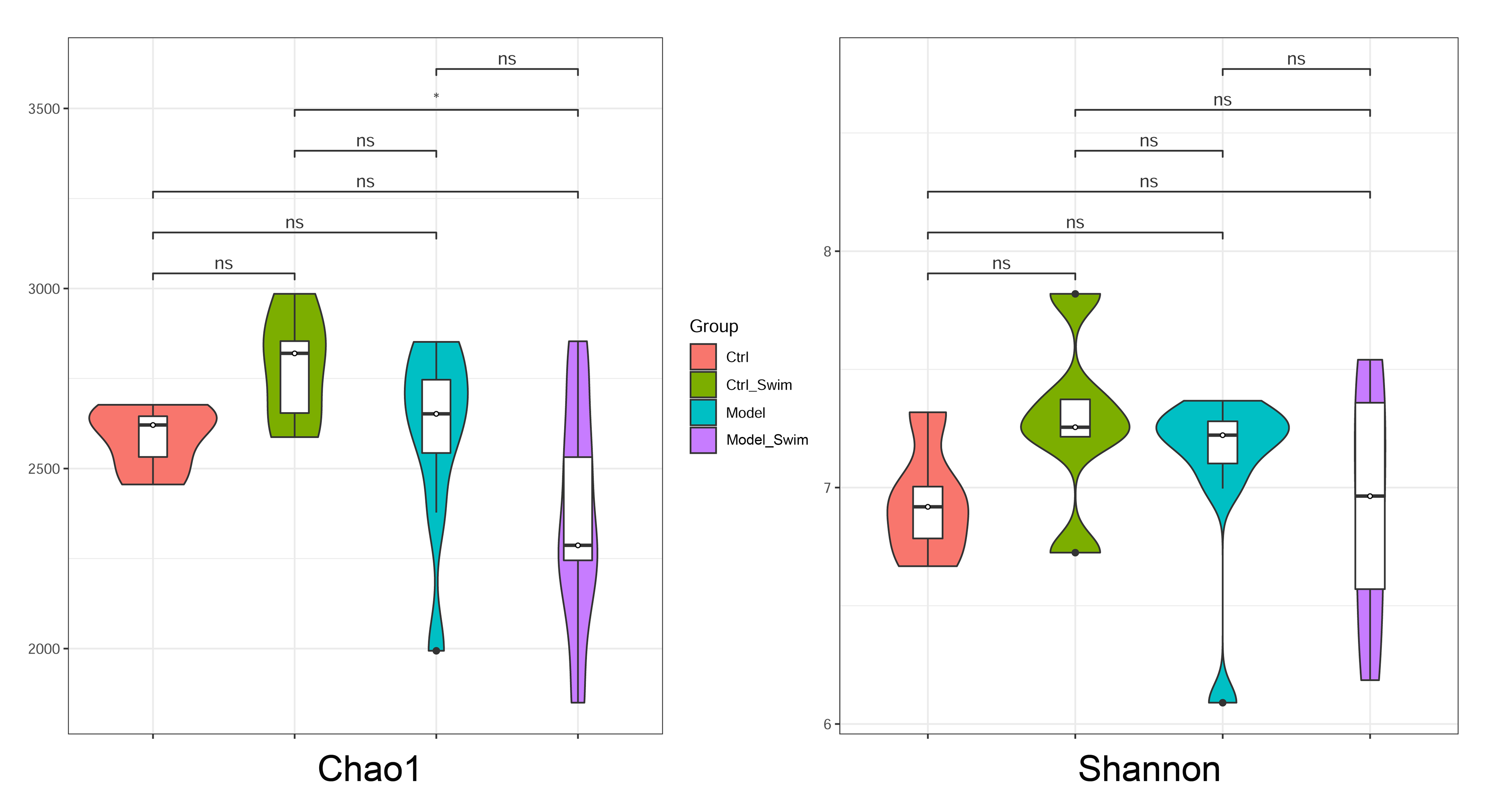

Supplement: Supplementary file 2 [file Image_2.tif]

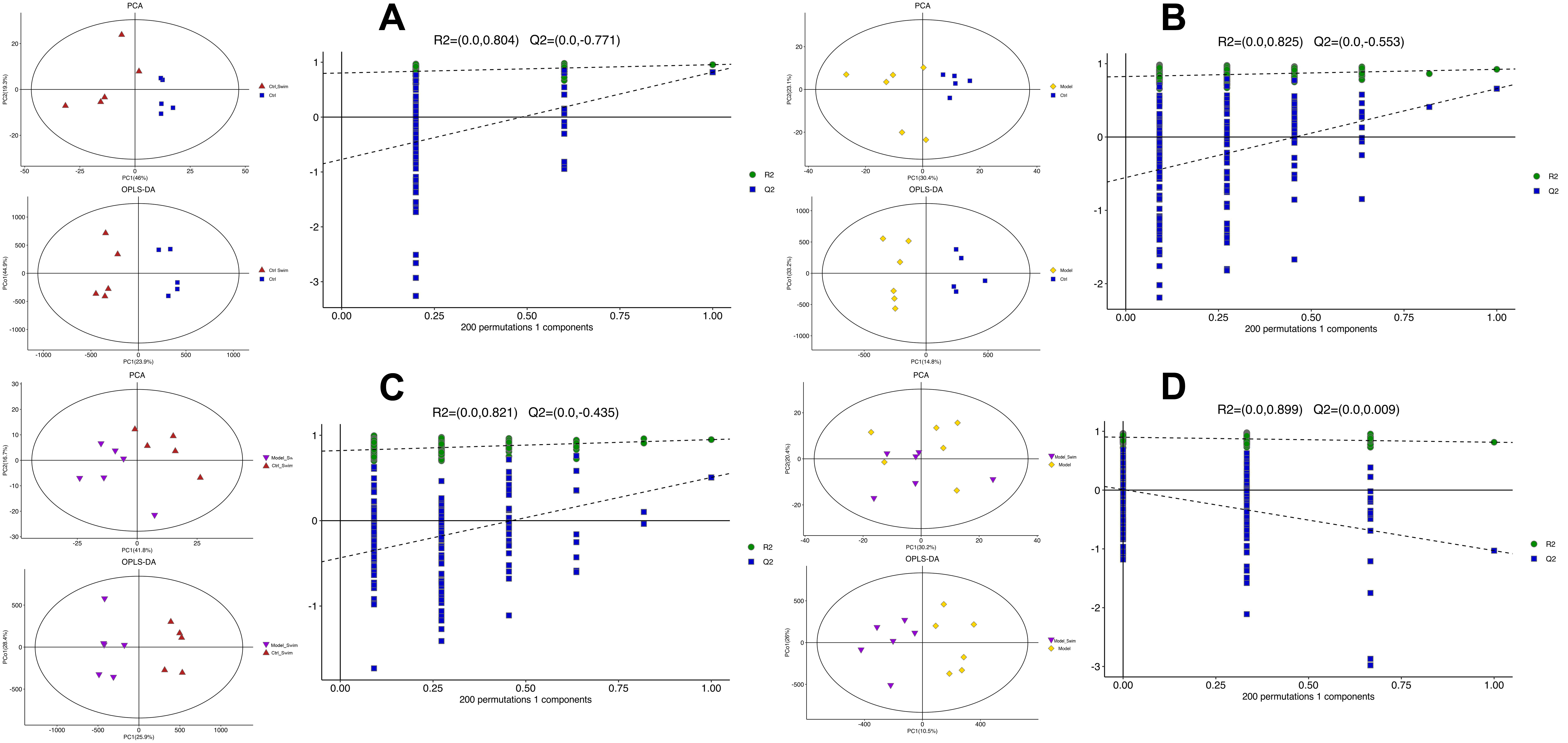

Supplement: Supplementary file 3 [file Image_3.tif]

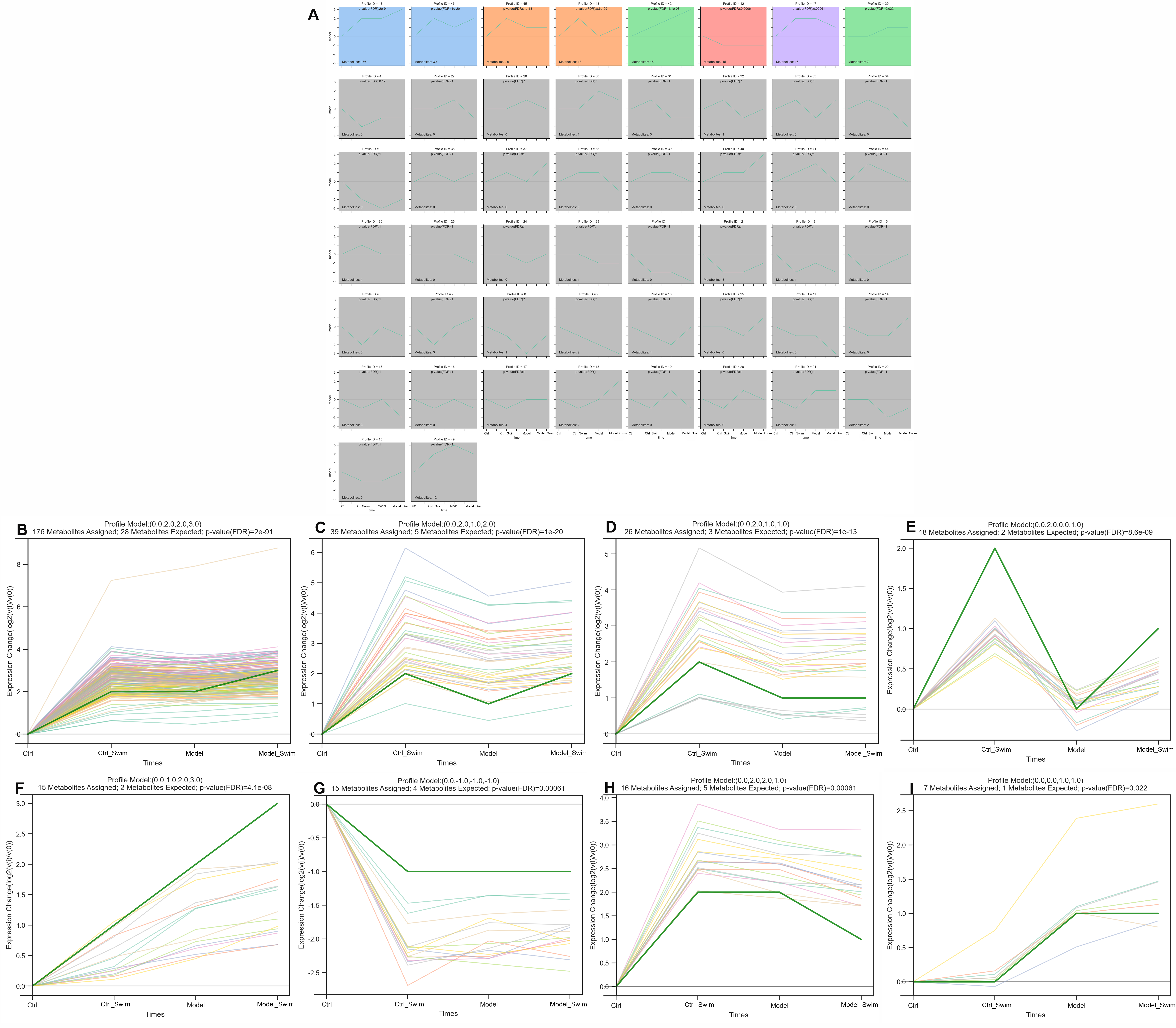

Supplement: Supplementary file 4 [file Image_4.tif]

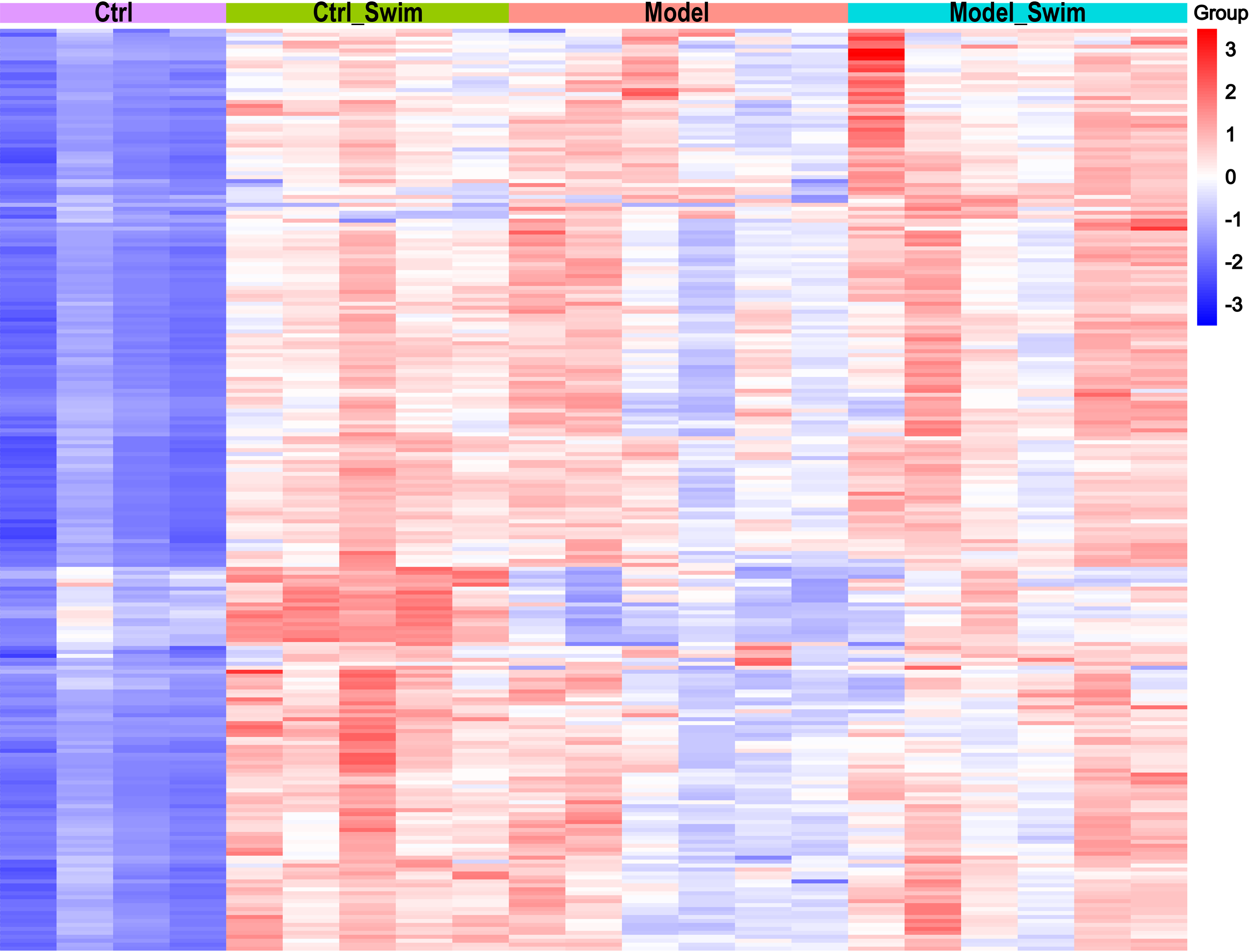

Supplement: Supplementary file 5 [file Image_5.tif]

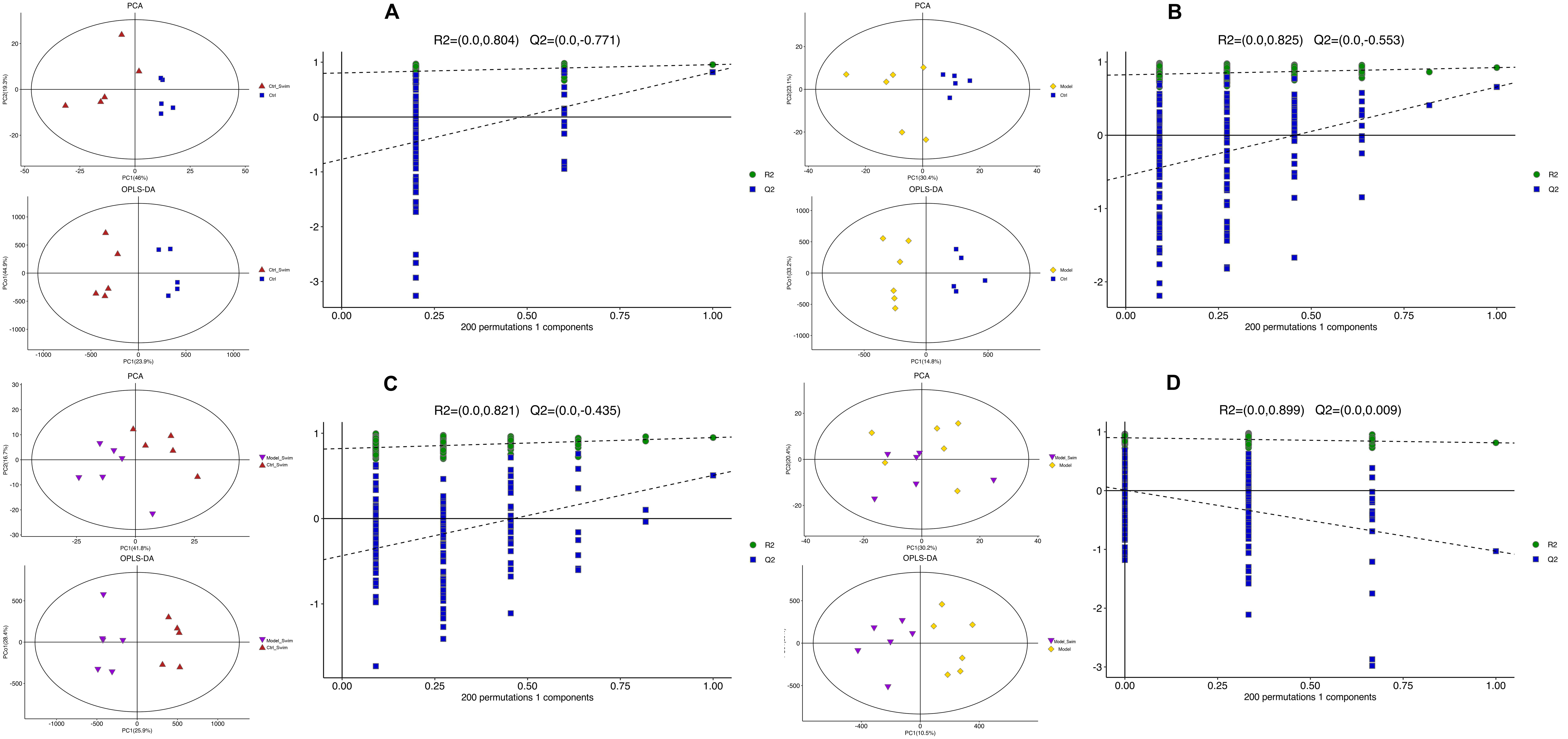

Supplement: Supplementary file 6 [file Image_6.tif]

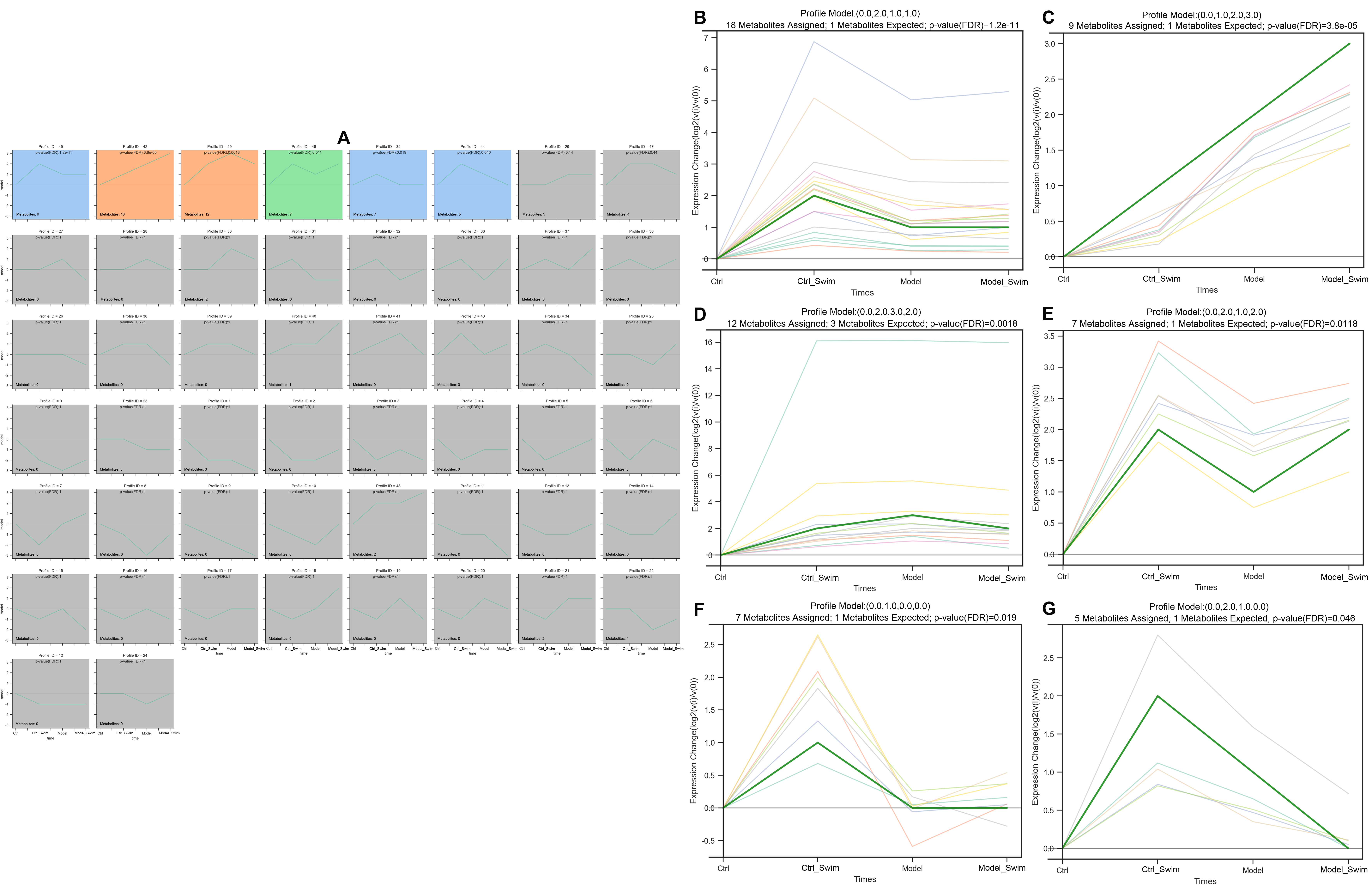

Supplement: Supplementary file 7 [file Image_7.tif]
