# Supplementary material for: Swimming Impedes Intestinal Microbiota and Lipid Metabolites of Tumorigenesis in Colitis-Associated Cancer
Source: Front Oncol. 2022 Jul 1;12:929092. doi: 10.3389/fonc.2022.929092 (PMC9285133; doi:10.3389/fonc.2022.929092)
Supplement: Supplementary file 8 [file Table_1.docx]

**Supplementary Table S1** Genera of bacteria changed in abundance among 4 groups.

| **Genera** | **p-value** | **FDR** | **Model_Swim**  **_mean** | **Model_mean** | **Ctrl_Swim**  **_mean** | **Ctrl_mean** |
| --- | --- | --- | --- | --- | --- | --- |
| Tyzzerella | 1.28E-05 | 0.003146 | 0 | 0 | 0.000139 | 0.000203 |
| Rikenellaceae | 5.69E-05 | 0.005741 | 1.74E-06 | 6.62E-06 | 0.00032 | 0.000138 |
| Faecalibaculum | 8.57E-05 | 0.005741 | 0.000141 | 0.002317 | 0.000544 | 0.000549 |
| RF39 | 0.000102 | 0.005741 | 1.41E-05 | 0.000166 | 6.51E-05 | 0.000827 |
| [Eubacterium]_ventriosum_group | 0.000117 | 0.005741 | 0 | 4.66E-05 | 0.00015 | 2.93E-06 |
| UCG-010 | 0.000206 | 0.008445 | 0.000249 | 0.000902 | 0.005046 | 0.002135 |
| Gordonibacter | 0.000312 | 0.010979 | 1.64E-06 | 0.000104 | 0.000147 | 0.000176 |
| [Eubacterium]_siraeum_group | 0.000378 | 0.011639 | 7.00E-06 | 1.14E-05 | 0.000136 | 0.00117 |
| Prevotellaceae_UCG-003 | 0.000528 | 0.01341 | 3.42E-06 | 0.000142 | 0 | 3.19E-06 |
| Anaerostipes | 0.000619 | 0.01341 | 3.75E-05 | 5.92E-05 | 0.013456 | 0.007308 |
| Bilophila | 0.000685 | 0.01341 | 1.53E-05 | 0.000221 | 0.000131 | 0.000195 |
| [Clostridium]_methylpentosum_group | 0.000693 | 0.01341 | 5.05E-06 | 1.82E-05 | 6.01E-05 | 0.000284 |
| Lachnospiraceae_UCG-001 | 0.000751 | 0.01341 | 1.98E-05 | 6.51E-06 | 0.000214 | 0.000375 |
| Mobiluncus | 0.000763 | 0.01341 | 1.64E-06 | 4.86E-06 | 1.61E-05 | 6.05E-05 |
| A2 | 0.00116 | 0.019019 | 1.06E-05 | 6.46E-05 | 0.000363 | 0.00024 |
| [Eubacterium]_fissicatena_group | 0.001974 | 0.030351 | 0.000409 | 0.002567 | 2.42E-05 | 0.000777 |
| Family_XIII_AD3011_group | 0.002391 | 0.033869 | 0.000191 | 0.000764 | 0.000715 | 0.0008 |
| Blautia | 0.002603 | 0.033869 | 0.000663 | 0.001298 | 0.000291 | 0.000398 |
| Anaerovorax | 0.002616 | 0.033869 | 0.000226 | 0.000456 | 0.001583 | 0.000505 |
| [Eubacterium]_coprostanoligenes_group | 0.004174 | 0.051339 | 0.000477 | 0.001049 | 0.001715 | 0.004641 |
| [Eubacterium]_xylanophilum_group | 0.004497 | 0.052676 | 0.003692 | 0.000644 | 0.007944 | 0.003522 |
| Allobaculum | 0.005018 | 0.053643 | 0.000923 | 0.001616 | 5.90E-06 | 2.99E-05 |
| UCG-007 | 0.005095 | 0.053643 | 1.52E-05 | 7.49E-05 | 0.000162 | 6.56E-05 |
| Staphylococcus | 0.005397 | 0.053643 | 0.000136 | 8.77E-05 | 0.000214 | 0.000375 |
| Bacteroides | 0.005452 | 0.053643 | 0.058279 | 0.022723 | 0.010682 | 0.007133 |
| Parabacteroides | 0.00656 | 0.06207 | 0.015652 | 0.007639 | 0.006095 | 0.002889 |
| Clostridium_sensu_stricto_1 | 0.006865 | 0.062546 | 0.000285 | 0.00065 | 0.000105 | 6.01E-06 |
| Coprobacter | 0.008021 | 0.066628 | 7.88E-06 | 0.000636 | 0 | 0 |
| Rikenellaceae_RC9_gut_group | 0.00812 | 0.066628 | 0.014563 | 0.00681 | 0.002781 | 0.001642 |
| UCG-009 | 0.008125 | 0.066628 | 0.000488 | 0.001318 | 0.001487 | 0.001373 |
| [Acetivibrio]_ethanolgignens_group | 0.009757 | 0.07445 | 0 | 3.34E-06 | 2.58E-05 | 3.90E-05 |
| Lachnospiraceae_UCG-004 | 0.009774 | 0.07445 | 0 | 0 | 1.24E-05 | 3.09E-06 |
| Atopobium | 0.009987 | 0.07445 | 0.000245 | 3.08E-05 | 0.000345 | 0.00013 |
| Erysipelatoclostridium | 0.011212 | 0.079432 | 0.004135 | 0.000586 | 0.000201 | 0.000279 |
| Monoglobus | 0.011301 | 0.079432 | 3.80E-05 | 2.38E-05 | 3.02E-05 | 0.000496 |
| Clostridia_UCG-014 | 0.012458 | 0.085129 | 0.014297 | 0.013547 | 0.021647 | 0.034178 |
| Lachnospiraceae_UCG-006 | 0.018324 | 0.121829 | 7.36E-05 | 0.000192 | 0.000309 | 0.000258 |
| Parasutterella | 0.023235 | 0.148151 | 0.032089 | 0.045272 | 0.006801 | 0.008568 |
| Gardnerella | 0.023717 | 0.148151 | 0.000446 | 2.17E-05 | 0.000553 | 0.000103 |
| Hirschia | 0.02409 | 0.148151 | 8.45E-06 | 0 | 0 | 0 |
| Acinetobacter | 0.026049 | 0.156297 | 6.99E-06 | 5.81E-05 | 1.55E-05 | 3.59E-05 |
| Papillibacter | 0.027619 | 0.161769 | 3.18E-05 | 0.00013 | 0 | 5.82E-05 |
| Lachnospiraceae_FCS020_group | 0.029056 | 0.165977 | 0.000134 | 0.000362 | 0.000309 | 0.000217 |
| DNF00809 | 0.029687 | 0.165977 | 1.59E-05 | 0 | 1.49E-05 | 6.11E-06 |
| Adlercreutzia | 0.032069 | 0.175311 | 2.46E-05 | 3.66E-05 | 1.60E-05 | 9.71E-05 |
| Marvinbryantia | 0.033369 | 0.178451 | 3.08E-05 | 2.21E-05 | 8.74E-05 | 0.000136 |
| Romboutsia | 0.035089 | 0.183659 | 0.000696 | 0.001138 | 0.000121 | 0.000469 |
| Lactococcus | 0.036896 | 0.189094 | 5.32E-06 | 2.56E-05 | 0 | 0 |
| Fastidiosipila | 0.038777 | 0.192088 | 1.03E-05 | 1.62E-06 | 1.84E-05 | 1.77E-05 |
| Rosenbergiella | 0.039042 | 0.192088 | 7.07E-06 | 1.62E-06 | 6.98E-06 | 2.07E-05 |
| Rikenella | 0.039932 | 0.192613 | 0.001481 | 0.003077 | 0.003286 | 0.003109 |
| Caulobacter | 0.042553 | 0.199119 | 2.64E-05 | 3.35E-06 | 2.83E-06 | 1.22E-05 |
| Demequina | 0.043525 | 0.199119 | 8.39E-06 | 1.72E-06 | 1.58E-05 | 0 |
| Halomonas | 0.044422 | 0.199119 | 1.03E-05 | 1.65E-05 | 2.72E-05 | 4.48E-05 |
| Roseovarius | 0.044519 | 0.199119 | 2.39E-05 | 4.31E-05 | 6.41E-05 | 1.20E-05 |

**FDR**, false discovery rate, represents the adjusted P value; **Model_Swim_mean**, the average abundance of value in Model_Swim group; **Model_mean**, the average abundance of value in Model group; **Ctrl_Swim_mean**, the average abundance of value in Ctrl_Swim group; **Ctrl_mean**, the average abundance of value in Ctrl group.
